# Perivascular Lymphocyte Clusters Induced by Gastric Subserous Layer Vaccination Mediate Optimal Immunity against Helicobacter through Facilitating Immune Cell Infiltration and Local Antibody Response

**DOI:** 10.1155/2020/1480281

**Published:** 2020-01-11

**Authors:** Chupeng Hu, Wei Liu, Ningyin Xu, An Huang, Zhiqin Zeng, Shuanghui Luo, Zhenxing Zhang, Menghui Fan, Feng Ye, Tao Xi, Yingying Xing

**Affiliations:** ^1^School of Life Science and Technology, China Pharmaceutical University, Nanjing 210009, China; ^2^Jiangsu Key Laboratory of Carcinogenesis and Intervention, China Pharmaceutical University, Nanjing 210009, China; ^3^Department of Gastroenterology, The First Affiliated Hospital of Nanjing Medical University, Nanjing, China

## Abstract

**Background:**

*In situ* vaccination-induced local inflammatory response resulted in the establishment of a pool of tissue-resident memory T (T_RM_) cells and new vessels after the resolution of inflammation. T_RM_ cells have received increasing attention; however, the role of new vessels in protective response is still unknown.

**Materials and Methods:**

We performed the laparotomy to access the stomach and injected alum-based vaccine into the gastric subserous layer (GSL). At 28 days post vaccination, a parabiosis mouse model along with depletion of anti-CD90.2 antibody was employed to explore the function of perivascular lymphocyte clusters in recall responses. The composition of the gastric lymphocyte clusters was analyzed by immunofluorescence staining. Antibody responses were detected using ELISA. Gastric lymphocytes were analyzed using flow cytometry.

**Results:**

GSL vaccination induced the formation of new vessels in the inflamed region. These new vessels were different from native vessels in that they were generally accompanied by perivascular lymphocyte clusters that mainly consisted of CD90-expressing cells. Additionally, histological analysis revealed the presence of CD4^+^ and CD8^+^ T cells in the perivascular lymphocyte clusters. Administration of a dose of an anti-CD90.2 antibody to GSL-vaccinated mice resolved these clusters. The efficacy of protection was compared in the parabiosis mice. Upon challenge, the presence of perivascular lymphocyte clusters was responsible for the fast recall response, as depletion of these clusters by CD90.2 antibody administration resulted in decreased expressions of VCAM-1, Madcam-1, and TNF-*α*, as well as lower recruitment of proinflammatory immune cells, decreased antibody levels, and poor protection.

**Conclusions:**

Our research demonstrates that *in situ* vaccination-induced regional inflammatory response contributes to optimal recall response not only by establishing a CD4^+^ T_RM_ pool but also by creating an “expressway,” i.e., perivascular lymphocyte cluster.

## 1. Introduction

*In situ* vaccination-induced local inflammatory response induced not only the establishment of a tissue-resident memory T (T_RM_) cell pool [1, 2] but also the formation of new vessels [3]. Circulating immune cells generally permeate across postcapillary venule and infiltrate into the mucosa to induce protection against mucosal pathogens [4]. It remained unclear whether these vessels played a role in protective response.

In a previous study, we used vaccine composed of aluminum adjuvant and CCF (CCF, a recombinant *Helicobacter pylori* subunit vaccine, a dual-antigen epitope, and a dual-adjuvant vaccine constructed as previously described [5]), to vaccinate the gastric subserous layer (GSL) of mice [6]. GSL vaccination-induced mucosal inflammation in the stomach and subsequently a pool of CD4^+^ tissue-resident T cells (CD4^+^ T_RM_) were established in the epithelium. After *in situ* injection with 5 *μ*L vaccine in the GSL, acute inflammation was observed at the mucosa around the vaccination site. Interestingly, upon challenge, circulating immune cells preferentially migrated towards the infected epithelium through vessels, which were located in the vaccination region (Supplementary Figure 1). It is noteworthy that new blood vessels are prevalent in inflammation-experienced tissue [1, 7, 8]. We speculated that they may play a role that distinguished them from the native vessels. Perivascular infiltration of DC-T cell clusters was formed at antigen-challenged sites, which enhanced proliferation and reactivation of effector T cells through integrin LFA-1 and local antigen exposure [9]. Additionally, perivascular infiltration of lymphocytes was associated with recruitment of myeloid immune cells and lymphocytes to peripheral tissue in a manner dependent on adhesion molecules or cytokines [9–11]. One of the patterns was classical inflammation-induced activation of endothelial cells through secreting VCAM-1 or Madcam-1 and various cytokines [12–14]. As described above, perivascular leukocyte clusters play an important role in the progression of inflammatory response. In this study, to highlight the need for *in situ* vaccination, we investigated the role of new vessels with perivascular lymphocyte clusters that were generated in *in situ* vaccination-induced inflammation in recall response.

## 2. Material and Method

### 2.1. Reagents

Alum adjuvant was purchased from Thermo Company. Fetal bovine serum (FBS) was purchased from Gibco Laboratories (Grand Island, NY, USA). ChamQ™ SYBR qPCR master mix (high ROX premixed) was obtained from Vazyme Biotech Co., Ltd. (Nanjing, China). 3,3,5,5-Tetramethylbenzidine (TMB) solution was purchased from Solarbio. TRIzol reagent was procured from Invitrogen. In vivo Mab anti-mouse CD90.2 and IgG_2b_ isotype were obtained from Bio X cell. All other solvents and chemicals were of analytical grade and used without further purification.

### 2.2. Vaccine Preparation

The preparation and storage of purified CCF protein, a dual-antigen epitope and dual-adjuvant vaccine constructed by cholera toxin B, multiepitopes from H. pylori urease, and self-adjuvant regions from S. typhimurium phase I flagellin FliC, called CTB-UE-CF (CCF), were performed in previous protocols [15]. Briefly, the CCF protein was expressed by Escherichia coli Rosetta (DE3) cells with pET-28a-CCF. The protein was first purified using nickel affinity chromatography (GE Healthcare), followed by anion-exchange chromatography with DEAE Sepharose FF [16] (Amersham Pharmacia Biotech AB, Sweden). The purity of CCF was confirmed using the Coomassie blue staining. Vaccine with alum was prepared with equal volumes of CCF solution and alum adjuvant.

### 2.3. Animals

Female 6 to 8-week-old C57BL/6 mice were purchased from the Comparative Medicine Center of Yangzhou University and housed under specific pathogen-free conditions in the Animal Experimental Center of China Pharmaceutical University. All animal experiments were approved by the Animal Ethical and Experimental Committee of the China Pharmaceutical University.

### 2.4. Gastric Subserous Layer Vaccination

The 6 to 8-week-old female C57BL/6 mice were anesthetized using intraperitoneal injection with a mixture of 100 mg/kg ketamine and 15 mg/kg xylazine; the experiment was conducted under aseptic conditions, and mice were placed on the thermostatic hot plate. After shaving off around the right abdomen, a 1 cm wide skin incision was made above the location of the stomach. After simple celiotomy laparotomy, the stomach was exposed using Forceps, and the gastric subserous layer of the greater curvature was injected with 5 *μ*L vaccine preparation using a microsyringe with a 33G needle. Next, the peritoneal incision was uninterruptedly sutured with 7-0 PGA absorbable suture, and the skin incision was performed using interrupted sutures (Shanghai Pudong Jinhuan Medical Products Co., Ltd.).

### 2.5. Parabiosis Mouse Pair Surgery

Mice were cohoused for 2 weeks prior to jointed surgery [17]. The experiment was performed under aseptic conditions. Naïve and immunized mice were anesthetized using intraperitoneal injection with a mixture of 100 mg/kg ketamine and 15 mg/kg xylazine. After shaving the corresponding lateral aspects of each mouse, a matching incision was made on the lateral skin from the elbow to the hip. Then, the tibia and ulna of each mouse were bound together with 3-0 PGA nonabsorbable suture (Shanghai Pudong Jinhuan Medical Products Co., Ltd.) to join the forelimbs and hind limbs together; the matching skin incision of each mouse was sutured together using 5-0 PGA absorbable suture (Shanghai Pudong Jinhuan Medical Products Co., Ltd.) to induce angiogenesis for a shared circulation between two mice.

### 2.6. Preparation of Single Cell Suspensions from Gastric Mucosa

The single cell suspensions were prepared similarly as previously described [15, 18]. In brief, the whole stomach of mice was cut and isolated using lesser curvature, prior to being removed; the content was placed into 15 mL RPMI1640 containing 10 mM HEPES, 10% PBS, 4 mM EDTA, and 0.5 mM DTT. Gastric epithelial lymphocytes were isolated by shaking at 250 rpm and 37°C for 30 min. The single cell suspension was obtained by passing it through a 70 *μ*m cell strainer. After washing and centrifugation, cell pellets were resuspended in an appropriate medium for further analysis or culture.

### 2.7. Preparation of Single Cell Suspensions from the Blood

The blood was isolated from mice and resuspended with 5 mL erythrocyte lysis buffer (BioLegend) and washed twice with 10 mL PBS containing 5% FBS. The cells were then collected for FACS analysis.

### 2.8. Antigen-Specific CD4^+^ T Cell Analysis

Antigen-specific CD4^+^ T cells were prepared as previously described [6]. Single cell suspensions from the stomach were purified with 67%/44% Percoll gradients. The cells at the interface were collected and washed with 7 mL RPMI 1640 containing 10% FBS. To detect Ag-specific CD4^+^ T cells, purified single cell suspensions from the stomach were stimulated for 12 h with 1∗10^6^ naïve CFSE-labeled splenocytes that were preloaded with CCF in RPMI 1640 containing 10% FBS and 5 *μ*g/mL Brefeldin A. After collection, the cells were stained for intracellular cytokines.

### 2.9. FACS Analysis

Single cell suspensions from the blood and gastric tissue were stained with the following antibodies: anti-CD45 (30-F11), anti-CD3*ε* (145-2C11), anti-CD90.2 (30-H12), anti-CD4 (GK1.5 or RM4-4), anti-CD11b (M1/70), anti-CD8*α* (53-6.7), anti-CD19 (6D5), anti-MHC class II (M5/114.15.2), anti-Ly6C (HK1.4), anti-Gr-1 (RB6-8C5), and anti-CD11c (N418) purchased from BioLegend or BD Pharmingen. All the samples were incubated for 20 min on ice in the dark. Multiparameter analyses were performed on a BD FACS Aria II or a BD FACSCalibur flow cytometer.

### 2.10. Immunofluorescent Staining

Frozen sections of gastric tissue (20 or 10 *μ*m) were cut and dried at room temperature. After blocking in a 5% bovine serum albumin PBS solution for 1 h, these sections were stained with the Abs an Alexa Fluor® 488-anti-CD4 (GK1.5, BioLegend) antibody and/or purified anti-CD11b (M1/70, BioLegend) or anti-CD8*α* (53-6.7, BioLegend), purified anti-mouse I-A/I-E (BioLegend), Alexa Fluor® 488-anti-CD90.2 (BioLegend), anti-CD31 (BD Biosciences), and anti-VCAM-1 (P3C4, BioLegend) antibody followed by goat anti-rat IgG2a/IgG2b Alexa Fluor® 488/594 antibody (BioLegend). The slides were washed, counterstained with DAPI to visualize cell nuclei, and analyzed with fluorescence microscopy. All images were acquired with a Panoramic 250 Flash III Scanner (3DHistech).

### 2.11. Quantification of Fluorescence Intensity

Fluorescence images were acquired with a Panoramic 250 Flash III Scanner (3DHistech); individual CD31 or VCAM-1 images were loaded into the ImageJ64 software as JPEG files. Color images were then converted into a binary scale, which transforms images into black and white pixels. Quantification was then performed on the black pixels using the “analyze particle” function. The mean fluorescence intensity in a random region was calculated.

### 2.12. Quantitative RT–PCR

Total RNA was extracted from gastric tissue using TRIzol reagent (Invitrogen), and each RNA sample was reverse-transcribed into cDNA using M-MLV (Promega, USA) and following standard protocols. Quantitative real-time PCR was performed with a conventional TaqMan SYBR green-based quantification method. The expressed genes in gastric tissues were quantitated using RT-qPCR and the set of primers that were designed by Sangon Biotech Co., Ltd. (Shanghai, China) based on the sequence numbers found in Supplementary Table 1.

### 2.13. Measurement of Antigen-Specific Antibody in the Serum

The blood was obtained from the mouse angular vein for the detection of the levels of antigen-specific antibody using ELISA as follows: the 96-well ELISA plates (Corning Laboratories, Corning, NY) were coated overnight at 4°C with 100 *μ*L of 10 *μ*g/mL CCF. 100 *μ*L of 1 : 100 diluted serum was added into the plates and then incubated at 37°C for 1 h. Then 100 *μ*L of diluted HRP-conjugated goat anti-mouse IgG1 and IgM (Santa Cruz, diluted 1 : 2000) was added as the second antibody and then incubated at 37°C for 1 h. The color reaction was performed through adding the substrate 3,3,5,5-tetramethylbenzidine (TMB) solution (Solarbio). The absorbance value was detected at 450 nm using a plate reader. Each sample was measured in triplicate.

### 2.14. Measurement of Antigen-Specific Secretory IgA Antibody in Gastric Tissue

The supernatant from the gastric tissue was obtained by homogenizing with a blade-blender homogenizer, and 100 *μ*L diluted supernatant in the proportion of 1 : 5 was added into the 96-well plates. HRP-conjugated goat anti-mouse IgA was performed to detect the sIgA. Other steps were carried out following the measurement of the antigen-specific antibody in the serum.

### 2.15. CD90.2^+^ T Cell Depletion Experiments

Six to eight-week-old female C57BL/6 mice were immunized through gastric subserous layer vaccination. After 4 weeks, each mouse was administered with a dose of 500 *μ*g anti-CD90.2 (30H12) or anti-RatIgG2b (LTF-2) antibody each mice i.p. 2 times at 3-day intervals [19]. In vivo depletion was confirmed through FACS analysis of the cell suspension from the blood.

### 2.16. *H. felis* Challenge

*H. felis* strain (ATCC 49179) was cultured, as previously described [20], at 37° Con interlayer of solid and liquid media both added with 7% heat-inactivated fetal bovine serum and 10 *μ*g/mL vancomycin under microaerophilic conditions. The bacteria were harvested through centrifugation and resuspended in fresh medium after 3-4 days. Prior to being inoculated with 0.5 × 10^9^ H*. felis* in 200 *μ*L medium, the mice were fasted 8 hours without solid food and 4 hours without water. All the groups were challenged 2 times at 3-day intervals via p.o. After 1 week, tissues from these mice were harvested and analyzed.

### 2.17. Measurement of *H. felis* in the Gastric Mucosa

To detect the colonization of *H. felis* in the gastric mucosa, qRT-PCR was performed to detect *H. felis* 16S expression in the gastric tissue. The total DNA of gastric tissue was extracted with a Qiagen DNA Tissue Mini Kit according to the manufacturer's instructions. Quantitation was performed with the ChamQ Universal SYBR qPCR master mix. RT-qPCR was performed using a set of primers and was designed by Sangon Biotech Co., Ltd. (Shanghai, China) based on the sequence numbers found in Supplementary Table 1. Analysis and fold change were determined using the CT method.

### 2.18. Statistics Analysis

The results are presented as the mean ± SD. Statistical analysis was performed using ANOVA or the *t*-test with the GraphPad Prism 6.0 software; values of *P* < 0.05 were considered a significant statistical difference for all experiments.

## 3. Result

### 3.1. Formation of New Vessels with Perivascular Clusters at Inflammation-Experienced Gastric Tissue

**Figure 1:**
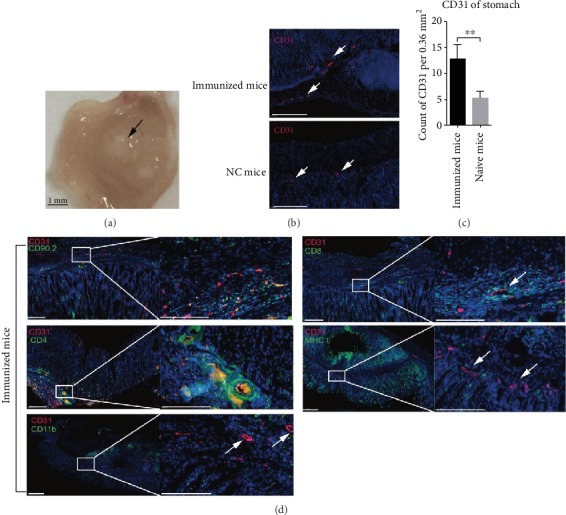
Formation of perivascular infiltrates of CD4/CD8^+^ T clusters in inflammation-experienced gastric subserous tissue. Six to eight-week-old female C57BL/6 mice were vaccinated in the gastric subserous layer. (a) 28 days later, the gastric tissue was exposed and a white lump was observed. (b, c) Frozen sections of gastric tissue from immunized or naïve mice were stained with antibodies against CD31 (red); nuclei are depicted by DAPI stained blue. Scale bars: 100 *μ*m. The fluorescence images were acquired with a Panoramic 250 Flash III Scanner (3DHistech); counts of CD31 per 0.36 mm^2^ were randomly chosen and calculated. Values are the mean ± SD (*n* = 3). ^∗∗^*P* < 0.01. (d) Frozen sections of gastric tissue from immunized mice were stained with antibodies against CD31 (red), CD90.2, CD4, CD8, CD11b, or MHCII (green); nuclei were depicted by DAPI stained blue. Scale bars: 200 *μ*m.

Gastric subserous layer (GSL) vaccination with alum-based vaccine induced mucosa inflammation in the stomach, and subsequently, a pool of CD4^+^ tissue-resident T cells (CD4^+^ T_RM_) was established in the epithelium. Upon challenge, we found that a large number of circulating immune cells preferentially migrated to the infected epithelium through vessels (Supplementary Figure 1), which were located at the vaccination region. To estimate the perivascular lymphocyte clusters formed in the vaccination region, we carried out an experiment: We injected alum-based vaccine into the GSL of mice. Then 28 days post immunization, the mice were sacrificed, and the gastric tissue was collected for further test. First, when the whole stomach was dissected, we found a white lump at the site of vaccination (Figure 1(a)). Next, we sniped longitudinally the vaccination region and performed immunofluorescence staining of CD31^+^ vessels. Substantially increased numbers of vessels were observed in the vaccination site of mice compared with those in the naïve mice (Figures 1(b) and 1(c)). Immunofluorescence staining indicated that abundant CD90.2 cells infiltrated around vessels in the vaccination region. Noteworthy histological assay showed perivascular infiltration of CD4^+^ T and CD8^+^ T cells, but not CD11b or MHCII-expressing cells emerged in the vessels of the vaccination region (Figure 1(d)). These findings led us to hypothesize that intensive accumulation of CD4^+^/CD8^+^ T cells around vessels of the vaccination region contributes to the formation of perivascular lymphocyte clusters.

### 3.2. Perivascular Lymphocyte Clusters Contributed to Protection against *H. felis* Infection

**Figure 2:**
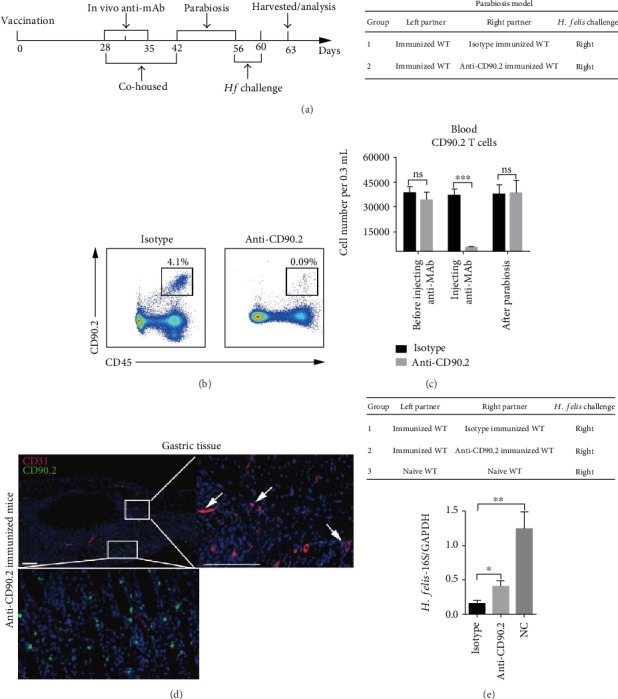
Perivascular lymphocyte clusters contributed to reduction of colonization of *H. felis* in gastric mucosa of mice. The depletion of perivascular lymphocyte clusters and a parabiosis mice model was performed as follows: (a) all mice were performed with laparotomy to access the stomach and injected with alum-based vaccine into GSL. At day 28, these immunized mice were divided into two groups. For one group, half of the mice were administered with anti-CD90.2 antibody and cohoused with the other half. At day 42, the anti-CD90.2 antibody-treated mice and the other mice not injected with an antibody were surgically jointed. At day 56, the indicated partner was infected with *H. felis*. Another group was treated the same way but used IgG2b isotype. After the later challenge on day 3, those mice were harvested and analyzed. (b, c) CD90.2 cells of the blood from indicated partner mice were analyzed by flow cytometry. Representative FACS plots were shown. (d) Frozen sections of gastric tissue from IgG2b isotype and CD90.2 antibody-treated mice were stained with antibodies against CD31 (red) and CD90.2 (green) and DAPI (blue). Scale bars: 200 *μ*m. (e) Mice were performed as indicated in the table; 3 days later, the mRNA level of *H. felis*-16S was detected by QRT-PCR. Values are the mean ± SD (*n* = 3). All indicated *P* values were tested using the ANOVA analyses or t tests. ^∗∗^*P* < 0.01; ^∗∗∗^*P* < 0.001; n.s.: not significant.

Next, we set out to characterize the role of the perivascular lymphocyte clusters in protective response. A parabiosis mouse model was established. All mice underwent laparotomy to access the stomach, and an alum-based vaccine was into GSL. At day 28, these immunized mice were divided into two groups. For one group, half of the mice were administered with anti-CD90.2 antibody and cohoused with the other half. At day 42, the anti-CD90.2 antibody-treated mice and the other mice not injected with an antibody were surgically jointed. At day 56, the indicated partner was infected with *H. felis* (Figure 2(a)). Another group was treated the same way, but the IgG2b isotype was used. The establishment of a parabiosis model is aimed at sharing circulating CD90.2 cells between the antibody-treated mice and immunized mice. As expected, compared to that in the isotype-treated mice, the CD90.2 cells were depleted in the blood of the anti-CD90.2 antibody-treated mice (Figure 2(b)). At 2 weeks post parabiosis, the number of circulating CD90.2 cells in the two groups of mice was similar (Figures 2(b) and 2(c)). Meanwhile, an immunolocalization assay was performed and the images indicated that the perivascular lymphocyte clusters were depleted (Figure 2(d)).

Next, the indicated mice were infected with *H. felis* (Figure 2(e)). Compared with the naïve mice, a significant decrease of *H. felis* in anti-CD90.2 or isotype control mAb-treated mice was detected by QRT-PCR. However, compared with isotype-treated mice, the colonization of *H. felis* in anti-CD90.2 antibody-treated mice was significantly higher (Figure 2(f)). These data suggested that perivascular lymphocyte clusters participated in protection against *H. felis*.

### 3.3. Perivascular Lymphocyte Clusters Accelerated Infiltration of Immune Cell in Gastric Mucosa

**Figure 3:**
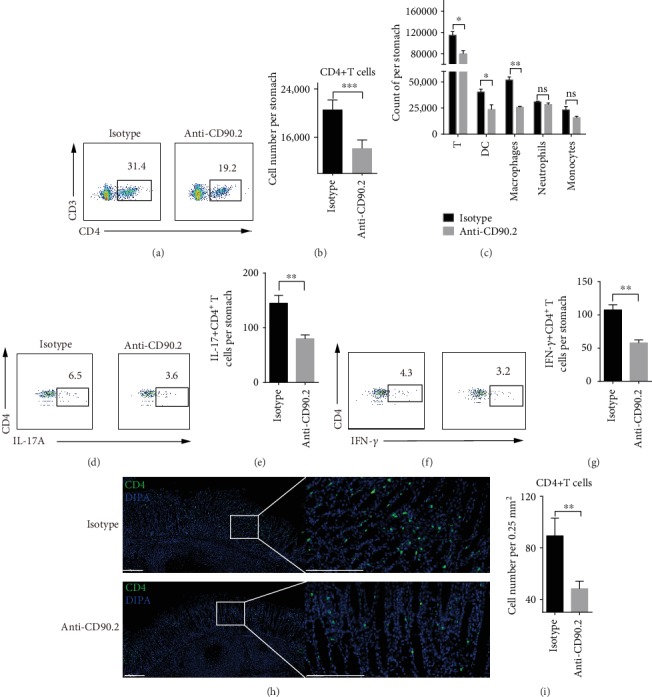
Perivascular lymphocyte clusters contributed to increased infiltrates of immune cells in gastric mucosa. Mice were administered as described in Figure 2(a) and the table of Figure 2(b). The single cells from gastric mucosa were analyzed by flow cytometry. The number of leukocytes (c), the percentage of total CD4^+^ T among CD3^+^ T cells, (a) and the number of total CD4^+^ T cells (b) were shown. (d–g) Single cells from gastric mucosa were stimulated with 1∗10^6^ naïve CFSE-labeled splenocytes that were preloaded with CCF, and then the percentage and number of IFN-*γ*/IL-17-producing CD4^+^ T cells were detected. (h) Frozen sections of gastric tissue from the indicated partner challenged with *H. felis* were stained with antibodies against CD4 (green), and nuclei were treated with DAPI (Blue). Scale bars indicated 200 *μ*m. (i) Counts of CD4 cells per 0.25 mm^2^ were randomly chosen and calculated. Values are the mean ± SD (*n* = 3). All indicated *P* values were tested using the ANOVA analyses or t tests. ^∗^*P* < 0.05; ^∗∗^*P* < 0.01; n.s.: not significant.

To explore how the perivascular lymphocyte clusters participated in protection against *H. felis*, we investigated the gastric mucosal infiltration of immune cells. We employed a parabiosis mouse model and anti-CD90.2 antibody depletion experiment as described above (Figure 2(a)). At day 3 post last infection, immune cell subsets of gastric mucosa were analyzed by flow cytometry. In comparison to those in the isotype-treated mice, most immune cell types except monocytes and neutrophils (i.e., total T cells, CD4^+^ T cells, DCs and macrophages) were prominently reduced in the anti-CD90.2 antibody-treated mice (Figures 3(a)–3(c)). The population of antigen-specific CD4^+^ T cells in gastric mucosa was analyzed using the flow cytometry. The results showed a significant decrease in IFN-*γ*and IL-17-producing CD4^+^ T cells in the anti-CD90.2 antibody-treated mice (3.2% and 3.6%, respectively) compared with those in the control mice (4.3% and 6.5%, respectively) (Figures 3(d)–3(g)). Similar results were observed in the immunofluorescence staining (Figures 3(h) and 3(i)). These data indicated that perivascular lymphocyte clusters contributed to mucosal infiltration of immune cells.

**Figure 4:**
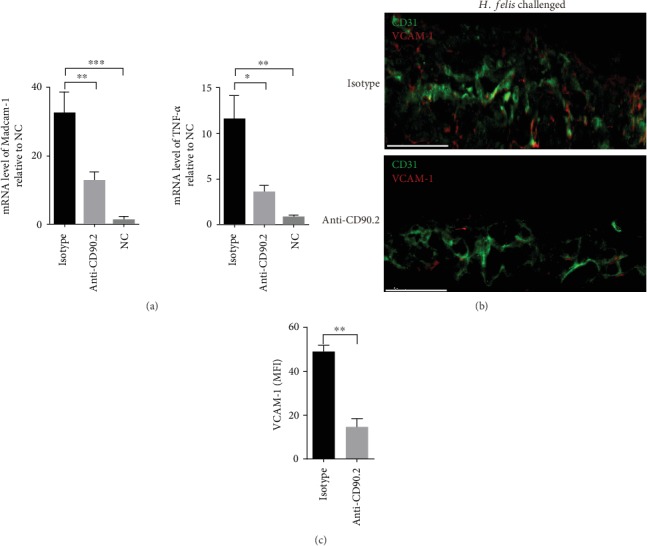
Perivascular lymphocyte clusters might increase the expression of VCAM-1, Madcam-1, and TNF-*α*. Mice were treated as indicated in Figure 2(a). (a) The expression level of Madcam-1 and TNF-*α* in the gastric tissue of the indicated partner mice challenged with *H. felis* was analyzed by qRT-PCR. (b) Frozen sections of gastric tissue from the indicated partner challenged with *H. felis* were stained with antibodies against CD31 (green) and VCAM-1 (red), and nuclei were treated with DAPI (blue). Scale bars indicated 50 *μ*m. (c) Mean fluorescence intensity (MFI) of VCAM-1 expression on CD31^+^ vessels was determined. Values are the mean ± SD (*n* = 3). All indicated *P* values were tested using the ANOVA analyses or t tests. ^∗∗^*P* < 0.01, ^∗∗∗^*P* < 0.001, and ^∗∗∗∗^*P* < 0.0001; n.s.: not significant.

In addition, at day 3 after the last infection, immunolocalization assay showed that anti-CD90.2 antibody-treated mice displayed a reduced expression of VCAM-1 in vessels of the vaccination region compared to that of the isotype-treated mice (Figures 4(b) and 4(c)). Similarly, the mRNA levels of Madcam-1 and TNF-*α* in the whole stomach of the anti-CD90.2 antibody-treated mice were reduced (Figure 4(a)). These data showed that perivascular lymphocyte clusters were associated with increased expression of VCAM-1 and Madcam-1 in the whole stomach, which might accelerate the recruitment of immune cells into the gastric mucosa.

### 3.4. Perivascular Lymphocyte Clusters Facilitated Antigen-Specific IgA Antibody Production

**Figure 5:**
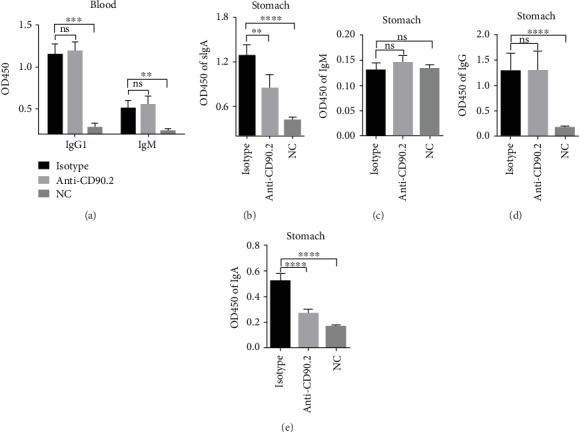
Perivascular lymphocyte clusters enhanced antigen-specific sIgA antibody response. All mice were performed with laparotomy to access the stomach and injected with alum-based vaccine into GSL. At day 28, these immunized mice were divided into two groups. For one group, half of the mice were administered with anti-CD90.2 antibody and cohoused with the other half. At day 42, the anti-CD90.2 antibody-treated mice and the other mice not injected with an antibody were surgically jointed. At day 56, the indicated partner was infected with *H. felis*. Another group was treated the same way but used IgG2b isotype. (a, b) After the later challenge on day 3, those mice were harvested and analyzed. The antigen-specific antibodies from the blood or gastric tissue were measured using ELISA. (c–e) After the later challenge at day 7, those mice were harvested and analyzed. The antigen-specific IgM, IgG, and IgA from gastric tissue were measured using ELISA. The absorbance value was detected at 450 nm using a plate reader. Values were the mean ± SD (*n* = 3). All indicated *P* values were tested using the ANOVA analyses or t tests. ^∗^*P* < 0.05, ^∗∗^*P* < 0.01, and ^∗∗∗^*P* < 0.001.

To assess whether perivascular lymphocyte clusters around vessels played a role in access of antigen-specific antibody to gastric tissue, we employed a parabiosis mouse model and anti-CD90.2 antibody depletion experiment as described above. The measurements of antibodies were performed on the whole stomach and blood. At 3 days after the last infection, ELISA revealed that levels of the IgM and IgG1 in the blood did not differ (Figure 5(a)), while antigen-specific IgA in the gastric tissue were reduced in the anti-CD90.2 antibody-treated mice compared with those in the isotype-treated mice (Figure 5(b)). After challenge at day 7, the antigen-specific IgM, IgG1, and IgA from gastric tissue were measured using ELISA. The result showed a higher expression of antigen-specific IgM, IgG1, and IgA in both anti-CD90.2 antibody-treated mice and isotype-treated mice compared with those in naive mice, while antigen-specific IgA in the gastric tissue were reduced in the anti-CD90.2 antibody-treated mice compared with those in the isotype-treated mice (Figures 5(c)–5(e)). Those results showed that antigen-specific IgA contributed to the early and later inflammation, but IgM and IgG1 participated in the later phase of gastric inflammation. Together, perivascular lymphocyte clusters were associated to infiltration of antigen-specific IgA antibody production in gastric tissue.

## 4. Discussion

Circulating immune cells (such as Th1/Th17 cells) permeate across postcapillary venules and infiltrate into the mucosa to induce protection against mucosal pathogens [4]. The role of Th1/Th17 cell-mediated post immunization gastritis is highlighted in the anti-*Helicobacter pylori* response [21, 22]. Our primary experiment found that new vessels with perivascular lymphocyte clusters emerged in the vaccination region after resolution of GSL vaccination-induced inflammation. Circulating immune cells preferentially migrated to the infected epithelium through vessels. Here, we employed a parabiosis mouse model and anti-CD90 antibody depletion experiment to explore the role of perivascular lymphocyte clusters in recall responses. Subsequently, we focused on the correlation between colonization of *H. felis* and perivascular lymphocyte clusters and demonstrated that perivascular lymphocyte clusters facilitated gastric mucosal infiltration of proinflammatory immune cells and sIgA antibody upon *H. felis* infection.

In our previous study, we have established an *in situ* vaccination mouse model that harbored a CD4^+^ T_RM_ pool in the stomach [6]. In the post vaccination acute phase, various immune cells were expanded and infiltrated into the vaccination region along with elevated levels of CXCL10 and CCL5 [6]. After resolution of inflammation, our data showed that abundant new vessels had emerged in the vaccinated region. Furthermore, we found that distinguished from native vessels, the new vessels were accompanied by a perivascular lymphocyte cluster. A recent study in the context of dermal perivascular leukocyte clusters suggests that a structure of perivascular DC-T cells clusters forms at the antigen-challenged site [9]. The DC clusters are followed by T cell accumulation around postcapillary venules and serve as the antigen-presentation sites for reactivation of effector T cells in the skin [9]. In comparison, we found in our models that CD4^+^ and CD8^+^ T cells rather than DCs were the main subsets present within the perivascular lymphocytes.

In the study, the parabiosis mouse model and anti-CD90.2 antibody depletion experiment demonstrated that depletion of perivascular lymphocyte clusters reduced the level of antigen-specific sIgA in the stomach, while no difference was seen in the level of IgG1 and IgM in the blood. The result suggested that perivascular lymphocyte clusters are related to mucosal sIgA response but not systemic antibody response. A recent research showed that CD4^+^ T cells help promote the access of antibody to neuronal tissue by triggering vascular permeability [23]. Proinflammatory cytokines act on endothelial cells to induce tight junction remodeling and increase permeability [24]. A similar result was observed, where the expression level of TNF-*α*correlated with perivascular lymphocyte clusters, which may facilitate mucosal infiltration of sIgA.

The stomach is a distinct organ that is inhabited by a very few native lymphocytes. Recruitment of circulating immune cells, such as Th1/Th17 cells and myeloid phagocytes, immediately is of utmost importance for inducing optimal anti-*H. pylori* response [22, 25, 26]. We found here that the perivascular lymphocyte clusters were “expressways” that allowed immune cell emigration into infected gastric mucosa. Depletion of perivascular lymphocyte clusters significantly reduced mucosal infiltration of DCs, macrophages, total T cells, and antigen-specific Th1/Th17 cells. Next, we investigated the correlation between migration of immune cells and perivascular lymphocyte clusters. Our data showed that mucosal infiltration of lymphocytes including total T cells and antigen-specific Th1/Th17 cells was expanded. It is noteworthy that lymphocytes generally permeate across the vessels by expressing VLA-4, which binds to endothelial VCAM-1 and alternatively spliced fibronectin (4). In a psoriatic skin, migration of lymphocytes into a psoriatic dermal skin is mediated by specific lymphocyte-dermal endothelial interactions [27]. This indicates that adhesion molecule or lymphocyte-endothelial interaction contributes to migration of lymphocytes. Consistent with the view that perivascular accumulation of lymphocytes is related to VCAM-1 expression in a perivascular adventitial fibroblast model [28], our data also demonstrated that the levels of expression of VCAM-1, Madcam-1, and TNF-*α* were attenuated in the absence of perivascular lymphocyte clusters. It is plausible that immune cells migrated through the endothelium by interaction between integrin ligands and adhesion molecules such as VCAM-1-*α*4*β*1 and Madcam-1-*α*4*β*7 [29, 30]. Meanwhile, recent studies found that local CD8^+^ T_RM_ do not only facilitate the access of circulating memory T cells to the site of virus reinfection in the genital tract but also secretes cytokines to trigger innate immunity in an IFN-*γ*-VCAM-1 axis [31, 32]. As described above, we proposed that these increased adhesion molecules and cytokines contributed to the emigration of immune cells. Furthermore, CD4^+^ T_RM_ might secrete cytokines to trigger the accumulation of perivascular lymphocyte clusters in the context of our mouse model [6]. Our future study would explore the correlation between CD4^+^ T_RM_ and perivascular lymphocyte clusters in the GSL-vaccination mouse model.

In some researches, an *in situ* immunization or infection mouse model was employed to explore the formation of T_RM_ after the resolution of immunization or infection-induced inflammatory responses [1, 33]. However, new vessels emerged after the resolution of local inflammation, but their role on immune responses often has not been given attention. Interestingly, our study indicated that *in situ* gastric vaccination-induced local inflammation induced not only the establishment of a CD4^+^ T_RM_ pool [6] but also the formation of new vessels with perivascular lymphocyte clusters, which contributed to mediating optimal immunity in recall response. These observations provide a new insight into the understanding of *in situ* vaccination-induced inflammatory response for the development of *H. pylori* vaccine. A concern is that GSL vaccination used in mice is not suitable for human, so our future study will look for a new approach to displace GSL vaccination. For example, a recent study shows that oral delivery of insulin targets the gastric mucosa by a self-orienting system [34]. This delivery system may be applicable for gastric mucosa-targeted vaccination.

## 5. Conclusions

The present study emphasized that *in situ* vaccination-induced proinflammatory response induced not only the establishment of a CD4^+^ T_RM_ pool but also the formation of perivascular lymphocyte cluster. Although the former provided prolonged protection, the latter also contributed to mediating optimal immunity by accelerating mucosal infiltration of immune cells and antibody response.

## Data Availability

The data used to support the findings of this study are available from the corresponding author upon request.

## Supplementary Materials

Supplementary Materials Supplementary Table 1: primer sequences of genes for quantitative RT-PCR assay. Supplementary Figure 1: immune cells emigrated towards the infected epithelium of the stomach through vessels in recall response.
