# Supplementary material for: Perivascular Lymphocyte Clusters Induced by Gastric Subserous Layer Vaccination Mediate Optimal Immunity against Helicobacter through Facilitating Immune Cell Infiltration and Local Antibody Response
Source: J Immunol Res. 2020 Jan 11;2020:1480281. doi: 10.1155/2020/1480281 (PMC7201474; doi:10.1155/2020/1480281)
Supplement: Supplementary Materials — Supplementary Table 1: primer sequences of genes for quantitative RT-PCR assay. Supplementary Figure 1: immune cells emigrated towards the infected epithelium of the stomach through vessels in recall response. [file 1480281.f1.doc]

Table 1

| name | 5’-3’ sequence | gen |
| --- | --- | --- |
| GAPDH-F | GAGCTGAACGGGAAGCTCAC | GAPDH |
| GAPDH-R | AGTCTAGCCCAAGATGCCCT |  |
| Madcam1-F | CCTGGCCCTAGTACCCTACC | Madcam1 |
| Madcam1-R | CCGTACAGAGAGGATACTGCTG |  |
| TNFα-F | GACGTGGAACTGGCAGAAGAG | TNFα |
| TNFα-R | TTGGTGGTTTGTGAGTGTGAG |  |
| *H. felis* 16S-F | CTAGCTTGCTAGGCGGATTAGT | 16S rRNA |
| *H. felis* 16S-R | CCTCACCAACAAGCTGATAGGA |  |

Primer sequences


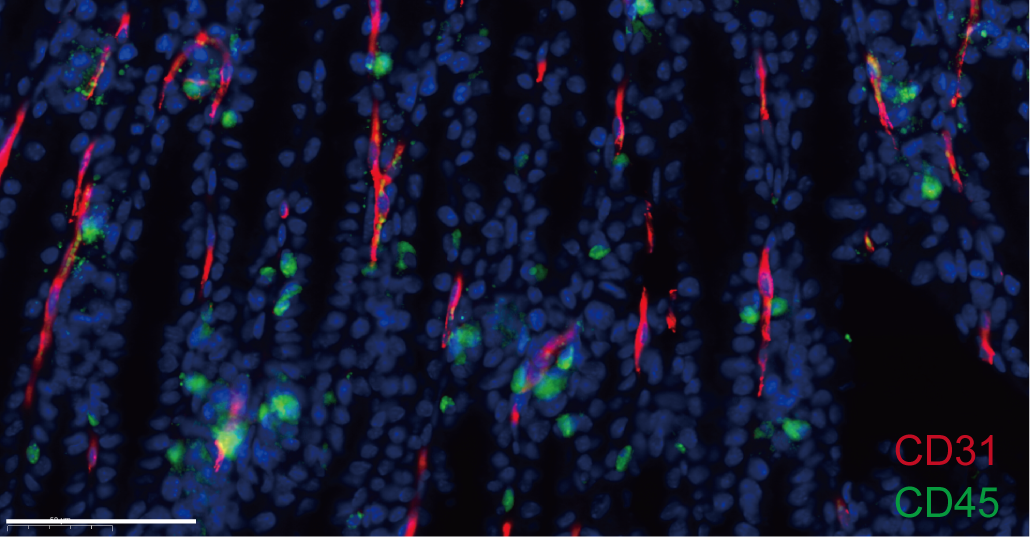


Figure 1 immune cells emigrated towards to infected epithelium through vessels in recall response.

6-8 week-old female C57BL/6 was vaccinated in the gastric subserous layer. 28 days later, those mice were infected with *H. felis* twice. Post 3 days last infection, those mice were sacrificed and stomach was collected for immunolocalization assay. Frozen sections of gastric tissue were stained with antibodies against CD31 (Red), CD45 (green), nuclei were depicted by DIPA stained blue, scale bars 100 µm.
